# *Sisymbriumlinifolium* and *Sisymbriopsisschugnana* (Brassicaceae), two new records from Xinjiang, China

**DOI:** 10.3897/phytokeys.119.32985

**Published:** 2019-03-15

**Authors:** Hongliang Chen, Ihsan A. Al-Shehbaz, Jipei Yue, Hang Sun

**Affiliations:** 1 CAS Key Laboratory for Plant Diversity and Biogeography of East Asia, Kunming Institute of Botany, Chinese Academy of Sciences, 132 Lanhei Road, Kunming, Yunnan 650201, China Kunming Institute of Botany, Chinese Academy of Sciences Kunming China; 2 University of Chinese Academy of Sciences, Beijing 100049, China University of Chinese Academy of Sciences Beijing China; 3 Missouri Botanical Garden, 4344 Shaw Boulevard, St. Louis, Missouri 63110, USA Missouri Botanical Garden St. Louis United States of America

**Keywords:** Brassicaceae (Cruciferae), China, new records, North America, *
Sisymbriopsis
*, *
Sisymbrium
*, Tajikistan

## Abstract

*Sisymbriumlinifolium* and *Sisymbriopsisschugnana*, previously confined to western North America and Tajikistan, respectively, were discovered in Xinjiang during a recent field trip to this autonomous region of China. The identity of these two species was subsequently confirmed by extensive morphological and molecular studies. The biogeographical significance of these new floristic records is briefly addressed.

## Introduction

Although mainly distributed in the temperate regions, species of the mustard family (Brassicaceae / Cruciferae; authorised alternative names, Art. 18.5 and 18.6 of the ICN: Turland et al. 2018) are found in all continents except Antarctica. In total there are 341 currently recognised genera, and about 4050 species worldwide (BrassiBase: https://brassibase.cos.uni-heidelberg.de, accessed 20 July 2018; Koch et al. 2012a, 2018, Kiefer et al. 2014, DA German pers. com.). For the Flora of China, 102 genera and 412 species were recorded by Zhou et al. (2001) but these numbers are out of date due to taxonomic status changes for some taxa and discoveries of new species. These include the reduction of *Desideria* Pamp., *Phaeonychium* O.E.Schulz, and *Eurycarpus* Botsch. to synonymy of *Solms-laubachia* Muschl. (Yue et al. 2008, German and Al-Shehbaz 2010), and the merging of *Neomartinella* Pilger, *Platycraspedum* O.E.Schulz, *Taphrospermum* C.A.Mey., and *Thellungiella* O.E.Schulz with *Eutrema* R.Br. (Al-Shehbaz and Warwick 2005, Al-Shehbaz et al. 2006). Besides, several new genera have since been proposed, including *Shangrilaia* Al-Shehbaz, J.P.Yue & H.Sun (Al-Shehbaz et al. 2004), *Metashangrilaia* Al-Shehbaz & D.A.German, *Rudolf-kamelinia* Al-Shehbaz & D.A.German, and *Anzhengxia* Al-Shehbaz & D.A.German (Al-Shehbaz and German 2016), *Shehbazia* D.A.German (German and Friesen 2014), *Sinoarabis* R.Karl, D.German, M.A.Koch & Al-Shehbaz (Koch et al. 2012b), *Sinalliaria* X.F. Jin, Y.Y.Zhou & H.W.Zhang (Zhou et al. 2014), as well as new species in *Solms-laubachia* (Yue et al. 2005, 2008, Chen et al. 2018a), *Draba* L. (Al-Shehbaz 2002, 2007, Al-Shehbaz et al. 2014), *Cardamine* L. (Al-Shehbaz and Boufford 2008, Al-Shehbaz 2015a, 2015b, 2015c), *Eutrema* (Gan QL and Li XW 2014, Xiao et al. 2015, Hao et al. 2015, 2016, 2017), and new records, i.e. *Cardaminebellidifolia* L. (Chen et al. 2011), *Pterygostemonspathulatus* (Kar. & Kir.) V.V.Botschantz. [reported as *Fibigiaspathulata* (Kar. & Kir.) B. Fedtsch. (German et al. 2012)], *Rhammatophyllumerysimoides* (Kar. & Kir.) Al-Shehbaz & O. Appel (German et al. 2006), and *Erysimumcroceum* Popov (Ya et al. 2018). According to our most recent compilation, there are 101 genera and 490 species of Brassicaceae in China.

**Figure 1.**
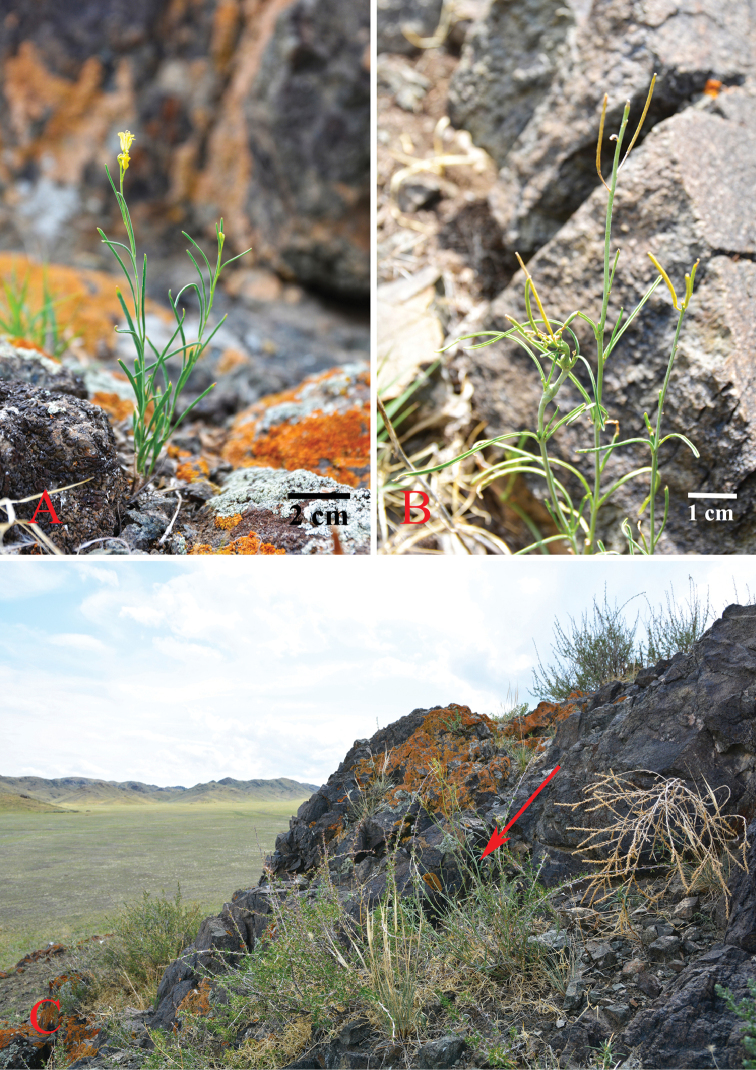
*Sisymbriumlinifolium* (Nutt.) Nutt. **A** flowering plant **B** fruits **C** habitat.

**Figure 2.**
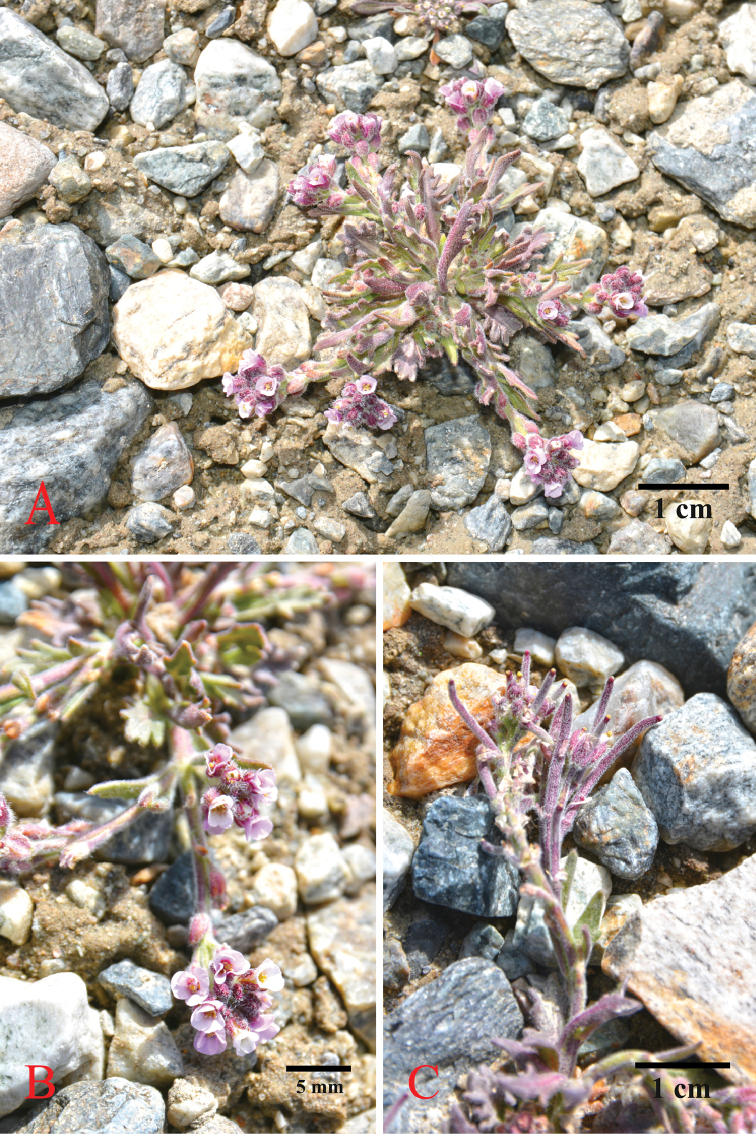
*Sisymbriopsisschugnana* Botsch. & Tzvelev. **A** flowering plant **B** flowers **C** immature fruits.

**Figure 3.**
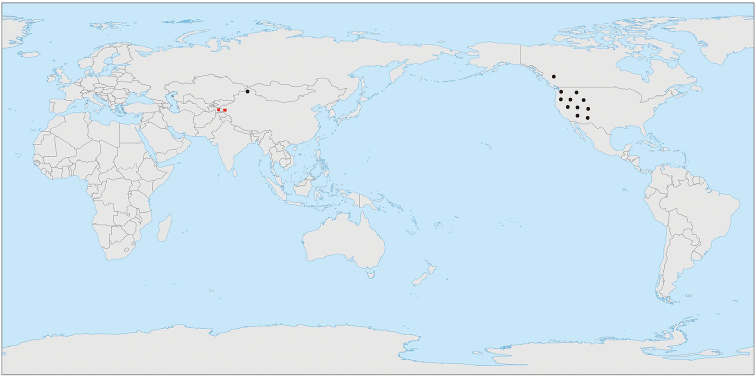
Distribution of *Sisymbriumlinifolium* and *Sisymbriopsisschugnana*. Black dots: *Sisymbriumlinifolium* in North America (modified from Al-Shehbaz et al. 2010) and the new population in Xinjiang, China; red squares: *Sisymbriopsisschugnana* in Tajikistan and Xinjiang, China.

Two of the authors (H.L.C and J.P.Y) have conducted a botanical expedition to Xinjiang and Xizang from 15 June to 22 July 2017, during which we collected about 130 species of 25 genera of Brassicaceae. Subsequent molecular and morphological studies supported the addition of two species as new records to China. *Sisymbriumlinifolium* (Nutt.) Nutt. (Figure 1) and *Sisymbriopsisschugnana* Botsch. & Tzvelev (Figure 2) were previously known only from western North America and Tajikistan, respectively (Figure 3).

## Materials and methods

### Plant materials and molecular data

Collected specimens were deposited in KUN, and species identification was based on the floras of China (Zhou et al. 2001), Pan-Himalaya (Al-Shehbaz 2015d), and North America (Al-Shehbaz et al. 2010) and studies on *Sisymbrium* L. (Al-Shehbaz 1988, 2006, Warwick and Al-Shehbaz 2003) and *Sisymbriopsis* (Al-Shehbaz et al. 1999).

The *nr*ITS sequence of *Sisymbriopsisschugnana* was included in our previous study on the phylogeny of the tribe Euclidieae (Chen et al. 2018b), while *nr*ITS sequences of four individuals of *Sisymbriumlinifolium* were generated and analysed in this study. An additional 48 sequences, representing 19 *Sisymbrium* species (mostly from Warwick et al. 2002) and seven sequences of five species (*Capsellabursa-pastoris* (L.) Medik., *Erucastrumsupinum* (L.) Al-Shehbaz & S.I.Warwick, *Neotorulariatorulosa* (Desf.) Hedge & J.Léonard, *Neuontobotryslanata* (Walp.) Al-Shehbaz, and *Polypsecadiumsolidagineum* (Triana & Planch.) Al-Shehbaz) were downloaded from GenBank (Appendix A). Following Mutlu and Karakuş (2015), *N.torulosa* and *C.bursa-pastoris* were used as outgroups.

### DNA extraction, PCR amplification, and sequencing

Total genomic DNA was extracted from silica gel-dried leaf materials using the Plant Genomic DNA Kit (Tiangen Biotech, Beijing, China) following the manufacturer’s protocol. The ITS region was amplified with the primers ITS-18F as modified by Mummenhoff et al. (1997) and ITS-4 (White et al. 1990). All polymerase chain reactions (PCR) were performed in a 25 μl volume consisting of 1μl sample DNA (approx. 5–10 ng), 12.5μl Premix Taq (Takara Biomedical Technology, Beijing, China), 1μl of 10 μM stock of each primer, adjusted to 25 μl with ddH_2_O. The PCR protocol of the ITS region involved a hot start with 4 min at 94 °C, and 32 cycles of amplification (1 min denaturing at 94 °C, 45 s annealing at 53 °C, 60 s extension at 72 °C), and a final elongation step for 10 min at 72 °C. The sequencing primers are the same as amplified primers.

### Phylogenetic analyses

Original chromatograms were evaluated with Sequencher 4.1.4 (Gene Codes Corporation, 2002) for base confirmation and contiguous sequences editing, and sequences were aligned with MAFFT v7.311 (Katoh et al. 2002; Katoh and Standley 2013) and were manually adjusted with MEGA 7.0.14 (Kumar et al. 2016). The aligned sequences were analysed with maximum parsimony (MP) and Bayesian Inference (BI).

Parsimony analysis was performed with heuristic searches of 1000 replicates with random stepwise addition using tree bisection reconnection (TBR) branch swapping as implemented in PAUP* 4.0a161 (Swofford 2018). All characters were weighted equally, and gaps were treated as missing data. For Bayesian Inference analysis, jModeltest v2.1.7 (Darriba et al. 2012) was used to select the best-fitted model of nucleotide substitution based on the Akaike information criterion (AIC), and the SYM+I+G model was selected for the ITS dataset. Bayesian Inference based on the Markov chain Monte Carlo methods (Yang and Rannala 1997) was performed using MrBayes v3.2.5 (Ronquist et al. 2012), four simultaneous Monte Carlo Markov chains (MCMCs) were run for 3 million generations, and one tree sampled every 1000 generations. The first 750 trees (25% of total trees) were discarded as burn-in. The remaining trees were summarised in a 50% majority-rule consensus tree, and the posterior probabilities (PP) were calculated.

## Results

The aligned ITS dataset comprised 24 species (59 accessions) with 584 characters, of which 192 were variable and 152 (26.03%) were parsimony-informative. Four individuals of the newly collected *Sisymbrium* from Xinjiang have exact sequences, and sequence divergence between them and *S.linifolium* ranged from 0–0.2%, which was lower than that of 1.5% compared with *S.polymorphum* (Murray) Roth.

**Figure 4.**
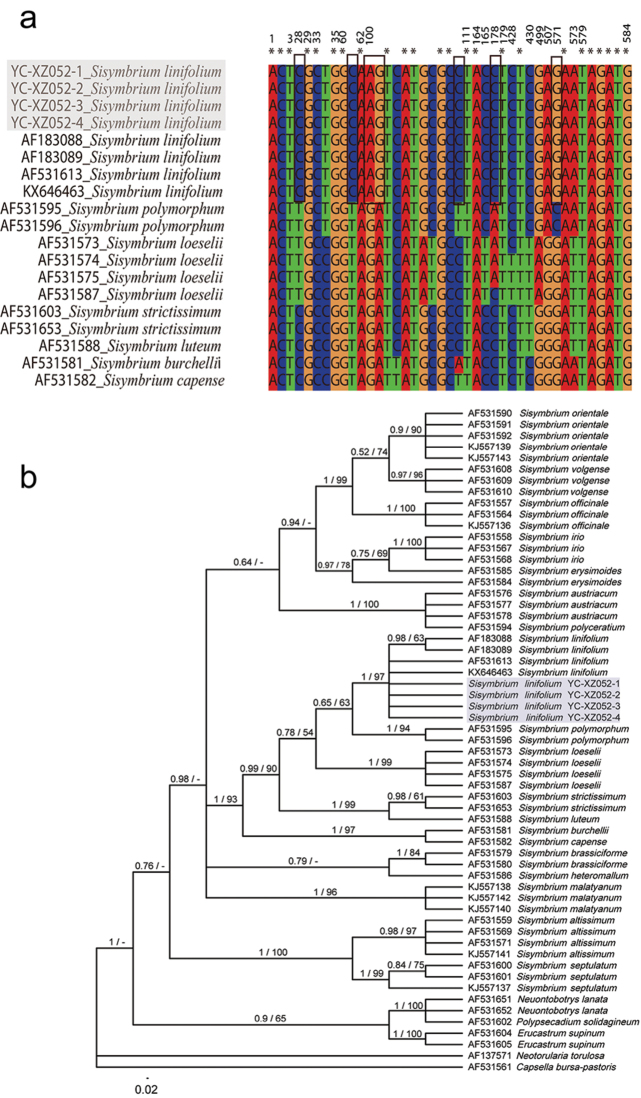
Multiple sequence alignment (**a**) and molecular phylogeny (**b**) based on ITS sequences. Bayesian posterior probability (PP) and MP bootstrap values (BS) are showed above branches in a following of PP / BS (only shown if > 50%). The newly found *Sisymbriumlinifolium* were in grey.

The generated MP trees had a very similar topology to the Bayesian tree, thus only the BI topology, which is almost as same as the result of Mutlu and Karakuş (2015), is shown. The four Xinjiang *Sisymbrium* clustered with *S.linifolium* (PP/BS = 1/97), and then clustered with *S.polymorphum* (PP/BS = 0.65/63) and *S.loeselii* L. (PP/BS = 0.78/54) (Figure 4b). Furthermore, sequences alignment revealed that the Xinjiang plants and North American *S.linifolium* shared several specific nucleotide residues that are different from *S.polymorphum* (Figure 4a), which further their identity as *S.linifolium*.

## Discussion

### *Sisymbriumlinifolium* (Nutt.) Nutt

The generic placement of *Sisymbriumlinifolium* has long been in dispute. It was originally placed in *Nasturtium* W.T.Aiton (Nuttall, 1834), and then transferred to *Sisymbrium* (Nuttall in Torrey and Gray, 1838) and *Schoenocrambe* Greene (Greene, 1896). Though several authors claimed that, on aspects of habit, leaves and flowers morphology, this species is very similar to the Eurasian *S.polymorphum* and retained it in *Sisymbrium* (Payson 1922, Schulz 1924, A1-Shehbaz 1973), while others kept it in *Schoenocrambe* (Rollins 1982, 1993). Molecular phylogenetic study on *Sisymbrium*, using ITS sequence data, revealed that *S.linifolium* is most closely related to *S.polymorphum* within the Old World *Sisymbrium* clade of tribe Sisymbrieae, while all other New World *Sisymbrium* were placed in various genera of the tribe Thelypodieae (Warwick et al. 2002). These results prompted Warwick and Al-Shehbaz (2003) to propose nomenclatural adjustments for some *Sisymbrium* species and further delimit *Sisymbrium* to include only 40 Old World species, plus North American *S.linifolium*, instead of the 96 species previously assigned to it (Al-Shehbaz, 2006).

Based on morphology, the Xinjiang *Sisymbrium* material we collected could be identified as *S.polymorphum*, but both phylogenetic analyses and sequence alignments supported its placement in *S.linifolium* (Figure 4). This conclusion makes the distribution range of *S.linifolium* extended from North America into north-western China, with a large range disjunction (Figure 3). One possible explanation for such distribution is a recent introduction of seeds of *S.linifolium* from North America to China by unintentional human activities. Many weeds of the mustard family (e.g., *Capsellabursa-pastoris*, *Thlaspiarvense* L., and *Sisymbriumorientale*) are invasive in both continents under preferable habitats (Zhou et al. 2007) such as farmlands, construction sites and ruins, waste places, disturbed sites, and roadsides. The Xinjiang *S.linifolium* was collected from a rocky hillside near the provincial road S229 in Jeminay County (Figure 1C). This locality is far from any villages or towns and, therefore, the possibility that its occurrence was the result of human activity is less likely. However, introduction with road construction material cannot be excluded as well.

Another possible explanation is that *Sisymbriumlinifolium* actually has both North American and Central Asian distribution, and most, if not all, of its Asian populations were misidentified as the very similar species, *S.polymorphum*. Further molecular phylogenetic studies and crossing experiments on more populations from both continents are needed to determine whether a single species or two are in fact involved. If it turned out that the species grows on both continents, then the name for the combined species should be the earlier-published one, *S.polymorphum*.

### *Sisymbriopsisschugnana* Botsch. & Tzvelev

*Sisymbriopsis* Botsch. & Tzvelev was originally recognised as a monospecific genus including *S.schugnana* as its type (Botschantsev and Tzvelev 1961). A second species, *S.mollipila* (Maxim.) Botsch., was transferred from *Sisymbrium* by Botschantsev (1966), and Al-Shehbaz et al. (1999) recognised three other species. Of the five species currently assigned to the genus, *S.schugnana* is endemic to Tajikistan, *S.mollipila* occurs in China, Kyrgyzstan, and Tajikistan, and the other three species are endemic to China. However, in a molecular phylogenetic study by Warwick et al. (2004), *S.mollipila* and *S.yechengensis* (C.H.An) Al-Shehbaz, C.H.An & G.Yang were found unrelated to each other, and the former was close to some *Neotorularia* species, whereas the position of *S.yechengensis* was unresolved. In a later phylogenetic study (Warwick et al. 2007), *S.mollipila* was found nested within a clade containing species of the genera *Desideria*, *Rhammatophyllum* O.E.Schulz, and *Solms-laubachia*, whereas *S.yechengensis* formed a solitary clade. Based on the distant genetic position and clear morphological differentiation, Al-Shehbaz and German (2016) transferred *S.yechengensis* to the new genus *Anzhengxia*.

The material studied here was collected from alluvium of the Muztagata (also Mugtag Ata) Glacial Public Park in Tashkurgan County, Xinjiang, an area close to the borders of Tajikistan. The plant has decumbent stems, dentate and palmately veined leaves, linear and latiseptate secund fruit, and white to pink flowers (Figure 2). Our initial morphological studies failed to identify the plant using Zhou et al. (2001), but subsequent molecular sequence comparison narrowed its identity to *Sisymbriopsis*, and its unique secund fruits led to its recognition as *S.schugnana* and a new record from China. In addition, species of *S.pamirica* (Y.C.Lan & C.H.An) Al-Shehbaz, C.H.An & G.Yang, *S.mollipila*, and *Anzhengxiayechengnica* (C.H.An) Al-Shehbaz & D.A. German were recently included in a phylogenetic study on the tribe Euclidieae (Chen et al. 2018b). Three *Sisymbriopsis* species formed a monophyletic subclade embedded in the *Solms-laubachia* s.l. clade, and *A.yechengnica* was close to *Pycnoplinthusuniflora* (Hook.f. & Thomson) O.E.Schulz., these findings suggesting that the real identity of *Sisymbriopsis* is still awaiting further studies (Chen et al. 2018b).

*Sisymbriopsisschugnana* is narrowly distributed in the eastern Pamir (Figure 3), a dry and cold desert plateau currently subjected to severe desertification caused by extensive exploitation of dwarf shrub resources, a phenomenon termed “Teresken Syndrome” (Kraudzun et al. 2014). Discovery of the first population of *S.schugnana* within the poorly explored Chinese mountains bordering Tajikistan should promote further botanical explorations in similar areas of adjacent neighbouring countries.

## Appendix A

Taxa and GenBank accession numbers for the nrITS sequences downloaded from GenBank and used in the phylogenetic analyses; an asterisk (*) indicates the new species record.

***Capsellabursa-pastoris*** (L.) Medik. (AF531561), ***Erucastrumsupinum*** (L.) Al-Shehbaz & S.I.Warwick (AF531604, AF531605), ***Neotorulariatorulosa*** (Desf.) Hedge & J.Léonard (AF137571), ***Neuontobotryslanata*** (Walp.) Al-Shehbaz (AF531651, AF531652), ***Polypsecadiumsolidagineum*** (Triana & Planch.) Al-Shehbaz (AF531602); ***Sisymbriumaltissimum*** L. (AF531559, AF531569, AF531571), ***S.austriacum*** Jacq. (AF531576, AF531577, AF531578), ***S.brassiciforme*** C.A.Mey. (AF531579, AF531580), ***S.burchellii*** DC. (AF531581), ***S.capense*** Thunb. (AF531582), ***S.erysimoides*** Desf. (AF531584, AF531585), ***S.heteromallum*** C.A.Mey. (AF531586), ***S.irio*** L. (AF531558, AF531567, AF531568), ***S.linifolium*** Nutt. (AF183088, AF183089, AF531613, KX646463), ***S.loeselii*** L. (AF531573, AF531574, AF531575, AF531587), ***S.luteum*** (Maxim.) O.E.Schulz (AF531588), ***S.malatyanum*** Mutlu & Karakuş (KJ557138, KJ557140, KJ557142), ***S.officinale*** (L.) Scop. (AF531557, AF531564, KJ557136), ***S.orientale*** L. (AF531590, AF531591, AF531592, KJ557139, KJ557143), ***S.polyceratium*** L. (AF531594), ***S.polymorphum*** (Murray) Roth (AF531595, AF531596), ***S.septulatum*** DC. (AF531600, AF531601, KJ557137), ***S.strictissimum*** L. (AF531603, AF531653), ***S.volgense*** M.Bieb. ex E.Fourn. (AF531608, AF531609, AF531610),****S.linifolium*** (Nutt.) Nutt. (MK419926, MK419927, MK419928, MK419929).
